# Impact of *Enterococci* vs. *Staphylococci* Induced Infective Endocarditis after Transcatheter Aortic Valve Implantation

**DOI:** 10.3390/jcm12051817

**Published:** 2023-02-24

**Authors:** Tomasz Gasior, Felix J. Woitek, Antonia Schroth, Mohamed Abdel-Wahab, Lisa Crusius, Stephan Haussig, Philipp Kiefer, Piotr Scislo, Zenon Huczek, Maciej Dabrowski, Adam Witkowski, Anna Olasinska-Wisniewska, Marek Grygier, Marcin Protasiewicz, Damian Hudziak, Utz Kappert, David Holzhey, Wojtek Wojakowski, Axel Linke, Norman Mangner

**Affiliations:** 1 Department of Internal Medicine and Cardiology, Herzzentrum Dresden, Technische Universität Dresden, 01307 Dresden, Germany; 2 Department of Cardiology, Heart Center Leipzig, University Hospital, 04289 Leipzig, Germany; 3 Department of Cardiac Surgery, Heart Center Leipzig, University Hospital, 04289 Leipzig, Germany; 4 1st Department of Cardiology, Medical University of Warsaw, 02-091 Warsaw, Poland; 5 Department of Interventional Cardiology and Angiology, National Institute of Cardiology, 04-628 Warsaw, Poland; 6 Department of Cardiac Surgery and Transplantology, Poznan University of Medical Sciences, 61-701 Poznan, Poland; 7 1st Department of Cardiology, Poznan University of Medical Sciences, 61-701 Poznan, Poland; 8 Institute of Heart Diseases, Wroclaw Medical University, ul. Borowska 213, 50-556 Wroclaw, Poland; 9 Department of Cardiac Surgery, Medical University of Silesia, 40-055 Katowice, Poland; 10 Department of Cardiac Surgery, Herzzentrum Dresden, Technische Universität Dresden, 01307 Dresden, Germany; 11 Department of Cardiac Surgery, Helios University Hospital Wuppertal, 42117 Wuppertal, Germany; 12 Department of Cardiology and Structural Heart Diseases, Medical University of Silesia, 40-055 Katowice, Poland

**Keywords:** TAVI, infective endocarditis, staphylococci, enterococci, prosthetic valve

## Abstract

Background: The two most common organisms found in infective endocarditis following transcatheter aortic valve implantation (TAVI-IE) are *enterococci* (EC-IE) and *staphylococci* (SC-IE). We aimed to compare clinical characteristics and outcomes of patients with EC-IE and SC-IE. Methods: TAVI-IE patients from 2007 to 2021 were included in this analysis. The 1-year mortality was the primary outcome measure of this retrospective multi-center analysis. Results: Out of 163 patients, 53 (32.5%) EC-IE and 69 (42.3%) SC-IE patients were included. Subjects were comparable with regard to age, sex, and clinically relevant baseline comorbidities. Symptoms at admission were not significantly different between groups, except for a lower risk for presenting with septic shock in EC-IE than SC-IE. Treatment was performed in 78% by antibiotics alone and in 22% of patients by surgery and antibiotics, with no significant differences between groups. The rate of any complication, in particular heart failure, renal failure, and septic shock during treatment for IE, was lower in EC-IE compared with SC-IE (*p* < 0.05). In-hospital (EC-IE: 36% vs. SC-IE: 56%, *p* = 0.035) and 1-year mortality (EC-IE: 51% vs. SC-IE: 70%, *p* = 0.009) were significantly lower in EC-IE compared with SC-IE. Conclusions: EC-IE, compared with SC-IE, was associated with a lower morbidity and mortality. However, absolute numbers are high, a finding that should trigger further research in appropriate perioperative antibiotic management and improvement of early IE diagnosis in the case of clinical suspicion.

## 1. Introduction

Over the past decade, transcatheter valve interventions have transformed the management of valvular heart disease, extending therapeutic options to patients of prohibitive or high surgical risk [1]. With an aging population, the demand for transcatheter aortic valve implantation (TAVI) is predicted to increase exponentially in the next years [2,3]. Simplified TAVI procedures with improved device design, along with shorter procedural times and early discharge strategies, all translated into low periprocedural complications and improved outcomes, with 1-year mortality ranging from 1% to 2% [4,5].

However, prosthetic valve endocarditis remains fatal unless treated appropriately. Infective endocarditis (IE) following transcatheter aortic valve implantation occurs in 0.7% to 3.4% of patients and is associated with poor prognosis, with a 1-year mortality of over 40% [6,7,8]. According to recent ACC/AHA guidelines, microbiological findings remain crucial in the diagnosis of IE and determining the clinical manifestation and treatment [9]. In many cases, the diagnosis of infective endocarditis can still pose a challenge and is frequently delayed, which may cause potentially irreversible structural damage to the heart and other organs secondary to thromboembolic or systemic immunologic reactions [10,11]. While *enterococci* and *staphylococci* are estimated to account for the majority of TAVI-IE cases, *enterococci* are often neglected in many antibiotic prophylaxis regimens [12]. Therefore, we aimed to compare baseline characteristics and outcomes of patients with *enterococci* (EC-IE) and *staphylococci* (SC-IE)-related TAVI-IE and sought to highlight the role and significance of the *enterococci* as a cause of IE.

## 2. Materials and Methods

### 2.1. Patient Cohort and Definitions

The registry included 163 patients with definite TAVI-IE diagnosed between 2007 and 2021 at 2 German and 5 Polish centers, with 53 (32.5%) and 69 (42.3%) patients having EC-IE and SC-IE, respectively. Each patient was retrospectively identified according to modified Duke criteria [12]. Informed consent for the procedure was obtained from all patients prior to TAVI intervention and the individual anonymized data sharing was performed according to the ethics committee of each center. Patients were included irrespective of the structure affected, which includes prosthetic or native valve, and cardiac implantable electronic devices. Based on microbiological findings, the cohort was divided into *enterococci* (EC) and *staphylococci* (SC) as a cause of IE. Early and late endocarditis were defined as the occurrence within the year and after a year following the procedure, respectively. The transcatheter aortic valve type was divided into balloon-expandable and self/mechanically-expanding. Perioperative mortality risk was defined according to the logistic EuroSCORE I [13].

### 2.2. Outcome Measures

The 1-year mortality was the primary outcome measure, while in-hospital death was a secondary outcome of the analysis. Clinical outcomes related to the TAVI procedure were defined according to VARC-2 criteria [14]. The complications during IE hospitalization were analyzed and included heart failure, acute renal failure, septic shock, stroke, systemic embolization, persistent bacteremia, and the composite of these complications. They were defined as follows: heart failure as a new onset or worsening of pre-existing chronic heart failure in accordance with the ESC guidelines [15]; acute renal failure defined as an increase in serum creatinine of ≥0.3 mg/dL (26.5 µmol/L) in 48 h or ≥1.5 fold increase in the last 7 days, or diuresis <0.5 mL/kg/h for 6 h following the VARC-2 definition [14]; septic shock following The Third International Consensus Definitions for Sepsis and Septic Shock (Sepsis-3) [16]; stroke as every new cerebral lesion with or without neurological symptoms in computed tomography and/or magnetic resonance imaging; systemic embolization as every new embolism in a peripheral organ (e.g., spleen or kidney) with or without clinical symptoms detected by appropriate imaging modalities; persistent bacteremia as defined as 3–7 days positive blood culture post-therapy.

### 2.3. Statistical Analysis

Data are presented as numbers and frequencies for categorical and as median (interquartile range, IQR) for continuous variables. Categorical variables were compared using the χ2 or Fisher’s exact test. Continuous variables were compared using the Mann–Whitney-U test after testing for variable distribution applying the Shapiro–Wilk test.

Estimates of all-cause mortality at 1 year were analyzed according to the method of Kaplan–Meier, and group comparisons were made applying the log-rank test. The independent association of EC-IE vs. SC-IE with 1-year all-cause mortality was determined with a Cox proportional hazard regression model, including age, sex, BMI, atrial fibrillation, and new pacemaker implantation.

Moreover, clinically relevant factors associated with the occurrence of EC-IE were evaluated using a binary logistic regression analysis. Clinically relevant factors showing a *p*-value ≤0.1 in univariate analysis were included in the multivariate model after excluding collinearity. Age and sex were forced into the model.

Furthermore, clinically relevant factors associated with 1-year all-cause mortality were evaluated within every group, applying a Cox proportional hazard regression model including parameters showing a *p*-value ≤ 0.1 in univariate analysis after excluding collinearity. Collinearity was assumed if R was greater than 0.70 in the bivariate correlation test, the tolerance-value was below 0.10, and/or the variable inflation factor (VIF) was greater than 10.

The statistical analysis was performed using SPSS Statistics version 27.0 (IBM Corporation, Armonk, NY, USA). A *p*-value < 0.05 was considered statistically significant.

## 3. Results

The registry included 163 patients with definite TAVI-IE according to the modified Duke criteria. Out of them, 53 (32.5%) *enterococci* (EC-IE) and 69 (42.3%) *staphylococci* (SC-IE) patients were included in this analysis. Baseline, peri-procedural characteristics, and in-hospital outcomes after TAVI are summarized in Table 1. Subjects were comparable with regard to age, sex, and most clinically relevant baseline comorbidities. However, a higher body mass index and higher rates of atrial fibrillation were evident in EC-IE, whereas new pacemaker (PM) implantation rates after TAVI were higher in SC-IE.

The main clinical characteristics, management, and outcomes of IE episodes are depicted in Table 2. Rates of early and late IE were comparable between *enterococci* and *staphylococci*. Symptoms on admission were not significantly different between groups, except a significantly lower risk of presenting with septic shock in EC-IE compared with SC-IE (EC-IE 15.4% vs. SC-IE 30.9%, *p* = 0.049).

Based on echocardiography, the vast majority of cases of EC-IE and SC-IE had evidence of typical IE vegetation (76.7%), as well as a considerable amount of perivalvular extension (15.6%). Vegetation size and rates of new aortic and mitral regurgitation were comparable between groups. TAVI prosthesis involvement was more likely in the EC-IE group (62.3% vs. 52.2%; *p* = 0.040), whereas the isolated PM vegetations were only found in SC-IE.

The rate of any complication, in particular heart failure, renal failure, and septic shock during treatment for IE, was lower in EC-IE compared with SC-IE (*p* < 0.05 for all comparisons). There were no significant differences between groups regarding in-hospital management. Overall, treatment was performed by antibiotics alone in 78.7% of all patients and a combined approach of antibiotics and surgery involved 22.1% of all cases, with no significant differences between groups.

**Figure 1:**
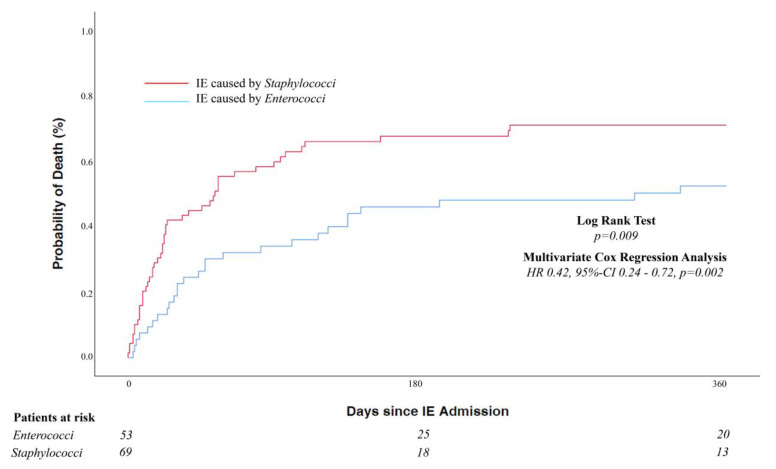
All-cause mortality according to IE caused by *enterococci* vs. *staphylococci*. HR indicates hazard ratio (adjusted by age, sex, BMI, atrial fibrillation, and new pacemaker implantation); CI, confidence interval; IE, infective endocarditis.

**Figure 2:**
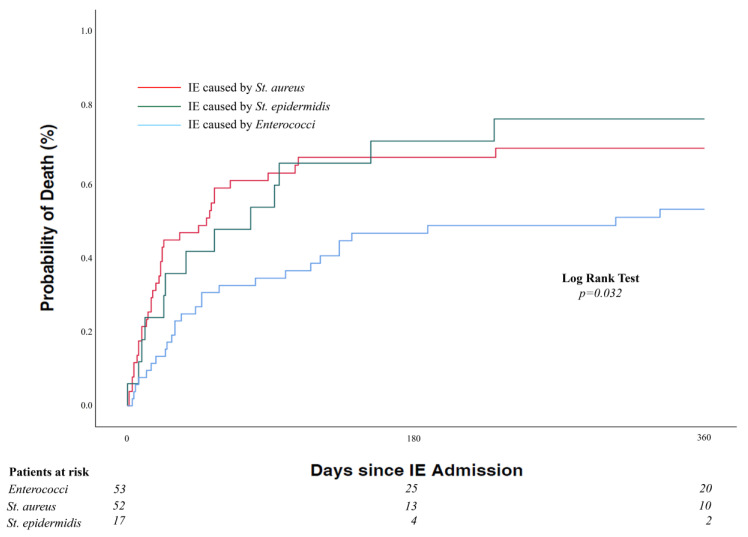
All-cause mortality according to IE caused by *enterococci* versus *St. aureus* versus coagulase-negative *St. epidermidis*.

In-hospital and 1-year mortality was dramatically high in both groups, however it was distinctly lower in EC-IE compared to SC-IE. In-hospital death occurred in 36.5% of cases of EC-IE and 55.9% of SC-IE (*p* = 0.035). Kaplan–Meier estimated rates of 1-year mortality were 50.9% and 69.6% in EC-IE and SC-IE, respectively (*p* = 0.009) (Figure 1). In a multivariate Cox proportional hazard model adjusting for age, sex, and baseline differences, EC-IE was associated with a significantly lower 1-year all-cause mortality (HR 0.42, 95%-CI 0.24–0.72, *p* = 0.002). Moreover, differentiating SC-IE into *St. aureus* and coagulase-negative *staphylococci* revealed a lower 1-year all-cause mortality in EC-IE compared to those two groups (Figure 2).

We have identified factors associated with EC-IE as shown in Table 3, including male gender (adjusted odds ratio (OR_adj_) 0.21, 95% confidence interval (CI) 0.04–1.00; *p* = 0.050), body mass index > median 27.3 kg/m^2^ (OR_adj_: 4.9, 95% CI 1.19–20.16; *p* = 0.028), atrial fibrillation (OR_adj_: 9.56, 95% CI 2.17–42.17; *p* = 0.003), new pacemaker implantation (OR_adj_: 0.09, 95% CI 0.01–0.65; *p* = 0.018), and septic shock on admission (OR_adj_: 0.12, 95% CI 0.02–0.71; *p* = 0.019).

Predictors of 1-year mortality of EC-IE are given in Table 4. Only the occurrence of septic shock during IE treatment remained independently associated with 1-year mortality in EC-IE (HR_adj_ 6.58, 1.51–28.66; *p* < 0.012).

Accordingly, as shown in Table 5, persistent bacteraemia was the only factor independently related to 1-year mortality in SC-IE (HR_adj_ 3.66, 1.03–13.02; *p* = 0.045). Cardiac surgery as a therapy decision was not significantly associated with increased 1-year mortality in both groups.

## 4. Discussion

We aimed to compare baseline characteristics and outcomes of patients with *enterococci* and *staphylococci*-associated TAVI-IE to highlight the importance of *Enterococcus* as a causative organism, which is frequently neglected in antibiotic prophylaxis regimens. The main findings of this analysis were: (1) EC-IE compared with SC-IE differed regarding initial symptoms and further course of the disease, which appeared to be less severe in EC-IE. (2) Among TAVI-IE, baseline characteristics like female gender, atrial fibrillation, and a body mass index >27.3 kg/m^2^ increased the susceptibility for EC-IE, whereas procedure- (new pacemaker implantation) and disease-related factors (septic shock on admission) were associated with a higher risk for SC-IE. (3) Both EC-IE and SC-IE were characterized by a high mortality; however, in-hospital and 1-year mortality were significantly lower in EC-IE compared with SC-IE.

Despite the evolution of TAVI over the years with simplified procedures and improved devices, the incidence of IE remains stable; the overall burden has increased in recent years due to the aging population and the number of patients referred to valve or other cardiac device interventions [17]. Although relatively rare, infective endocarditis after TAVI poses a life-threatening risk for patients with mortality rates over 40% in the first year [7,8] and a long-term prognosis that is worse than many malignances [6,12,18]. Microbiology reveals *staphylococci* or *enterococci* in roughly every second patient, making those bacteria the two most common organisms causing TAVI-IE [7,8,19,20]. *Enterococci* are 2–3 times more prevalent among patients with IE after TAVI compared to SAVR-related endocarditis [21]. This might be related to the clinical profile of TAVI patients representing a cohort of older patients with a relevant number of comorbidities and a more complicated and recurrent in-hospital treatment including the use of intravascular catheter, urinary catheter and nasogastric tube and use of antibiotics [22]. This is supported by the profile of our patients regardless of the causing organism presenting with at least one chronic condition (obesity, atrial fibrillation, chronic kidney disease), posing an increased risk of complications such as acute renal failure, persistent infection, heart failure, or in-hospital death. It also highlights the need for adequate prevention and disease control in patients after TAVI or other cardiac interventions involving implantable devices. As such, we identified patients receiving a new pacemaker implantation after TAVI as being at increased risk for SC-IE.

Moreover, the common femoral artery represents the preferred access in the vast majority of TAVI procedures. At the same time, common enterococcal colonization of the groin, even after routine disinfection before a TAVI procedure, might predispose to a high incidence of EC-IE [23]. Additionally, enterococcal colonization was associated with patients’ characteristics such as advanced age and comorbidities and was found to be more prevalent in obese people, which corresponds to our findings of high BMI (>27.3 kg/m^2^) as a significant factor related to EC-IE.

It is worth noting that all patients in our study were subjected to peri-procedural prophylaxis with cephalosporins, which could lead to insufficient protection against *enterococci* due to intrinsic resistance to cephalosporins. A high prevalence of EC-IE, and the fact that most prophylactic antibiotic regimens utilize cephalosporins before TAVI, highlight the need to study adapted antibiotic prophylaxis regimes with activity against *enterococci*, in particular in patients at increased risk for EC-IE, e.g., older females with a high BMI [24].

Initial symptoms and the clinical course of EC-IE compared with SC-IE appear to be less severe with a lower rate of septic shock on admission and lesser, yet still high complications during the treatment for IE. This might have translated into the lower in-hospital and 1-year mortality observed in our study. The increased mortality risk in IE caused by *staphylococci* is well established in native valve IE [12], IE of surgical bioprostheses [21], and in TAVI-IE [19]. It is important to mention that this lower mortality in EC-IE was observed despite no significant difference in perivalvular complications and the inclusion of seven isolated pacemaker infections SC-IE. In particular, perivalvular complications are known to be associated with a worse outcome [25]. It was previously described that it is not the method of treatment (surgery or antibiotics alone) that determines the outcomes of patients with TAVI-IE, as the high mortality of these patients was strongly linked rather to patients’ characteristics, pathogen, and IE-related complications [26,27]. In fact, the comorbidities and symptoms, both on admission and during the course of the disease, need to have higher awareness around them. This was also confirmed in our analysis with septic shock on admission and persistent bacteremia as independent predictors of 1-year all-cause mortality in EC-IE and SC-IE, respectively. Overall, these findings indicate that the virulence of the organism and the immune response of the host are important determinants of outcome.

Therefore, early diagnosis of IE is both important and challenging, yet critical in disease management, as a delay in the treatment process is associated with worse clinical outcomes [28]. Unfortunately, our registry did not collect the time from initial symptoms to diagnosis of TAVI-IE. The two mainstays of IE diagnosis are microbiological and imaging studies [12]. Despite the advancements in diagnostics, blood-culture-negative infective endocarditis (BCNIE) is reported in up to 31% of all IE cases, in particular after antibiotic therapy prior to blood culture sampling [12]. Furthermore, blood culture findings are not always a sufficient indicator for the actual bacteria found in the valve or perivalvular structures [29]. According to several studies, BCNIE poses a significant diagnostic and therapeutic challenge; however, it is associated with similar outcomes as blood-culture-positive infective endocarditis [30,31].

On the other hand, the absence of echocardiographic signs of endocarditis may lead to further delays in the appropriate treatment initiation. It has been described that patients with TAVI-IE and negative echocardiographic signs are at the same risk of in-hospital and 1-year mortality as patients with positive imaging [32]. Those findings implicate that the recommended diagnostic pathway and “Endocarditis Team”, as requested by the guidelines [12], needs to be implemented and provides an opportunity to improve patient outcomes [33].

## 5. Limitations

Several limitations should be considered while interpreting these results. First, this registry is observational, voluntary, and non-randomized, with the limitations and potential bias on data collection and analysis inherent to this setting. Second, there was no monitoring to verify the accuracy of data reported by each center. Third, despite the multicenter setting, the number of patients is still low, especially for differentiating SC-IE into *St. aureus* and coagulase-negative *staphylococci*. Fourth, most of the patients were at increased surgical risk before TAVI, and the operative risk was even higher after they developed IE. Therefore, projecting the future expansion of TAVI to younger, lower risk patients with less comorbidities, these results may not necessarily be transferable. Fifth, reflecting the hypothesis generating character of our analysis, no adjustment for multiple testing or analyzing competing risk was performed.

## 6. Conclusions

TAVI-IE remains a serious condition with poor outcomes and mortality. *Enterococci* and *staphylococci* are the most common microbiological findings in TAVI-IE. Despite a lower morbidity and mortality in EC-IE compared with SC-IE, the absolute event rates are high and should trigger further research in appropriate antibiotic management before TAVI procedures and improvement of early diagnosis in case of clinical suspicion of IE.

## Figures and Tables

**Table 1:** Baseline characteristics.

	All Patients (n = 122)	EC-IE (n = 53)	SC-IE (n = 69)	Unadjusted *p*-Value ^a^
**Baseline characteristics**				
Age, years, median (IQR)	80 (76–84)	80 (78–84)	81 (75–84)	0.396
Gender, male, n (%)	72/122 (59.0)	28/53 (52.8)	44/69 (63.8)	0.223
Body mass index, kg/m^2^, median (IQR)	27.3 (24.2–31.2)	29.0 (25.4–32.6)	25.8 (23.9–29.7)	0.029
Diabetes mellitus, n (%)	51/122 (41.8)	26/53 (49.1)	25/69 (36.2)	0.155
Atrial fibrillation, n (%)	57/122 (46.7)	32/53 (60.4)	25/69 (36.2)	0.008
Chronic kidney disease, n (%)	75/122 (61.5)	30/53 (56.6)	45/69 (65.2)	0.333
COPD, n (%)	34/122 (27.9)	16/53 (30.2)	18/69 (26.1)	0.616
Previous Stroke, n (%)	10/122 (8.2)	6/53 (11.3)	4/69 (5.8)	0.328
Previous heart surgery, n (%)	19/122 (15.6)	9/53 (17.0)	10/69 (14.5)	0.707
Previous infective endocarditis, n (%)	1/122 (0.8)	0/53 (0)	1/69 (1.4)	1.000
Logistic EuroSCORE, % median (IQR)	12.2 (7.2–21.5) n = 116	13.5 (7.9–24.1) n = 50	11.6 (6.5–19.1) n = 66	0.283
Left ventricular ejection fraction, % median (IQR)	56 (45–65) n = 120	59 (48–65) n = 52	55 (42–65) n = 68	0.135
Mean transaortic gradient, median (IQR), mmHg	43 (31–54) n = 112	46 (40–57) n = 46	40 (27–50) n = 66	0.001
Mitral regurgitation ≥ 2, n (%)	18/120 (15.0)	7/52 (13.5)	11/68 (16.2)	0.680
**Periprocedural characteristics**				
Implantation site				
Hybrid room, n (%)	122/122 (100)	53/53 (100)	69/69 (100)	N/A
Orotracheal intubation, n (%)	47/120 (39.2)	16/52 (30.8)	31/68 (45.6)	0.099
Antibiotic prophylaxis				
Cephalosporins alone, n (%)	102/102 (100)	45/45 (100)	57/57 (100)	n.a.
Approach, n (%)				
Transfemoral	116/122 (95.1)	51/53 (96.2)	65/69 (94.2)	0.696
Prosthesis type				
Balloon-expandable, n (%)	32/122 (26.2)	13/53 (24.5)	19/69 (27.5)	0.708
Self-/Mechanically expanding, n (%)	90/122 (73.8)	40/53 (75.5)	50/69 (72.5)	
**In-hospital Outcomes (TAVI)**				
Stroke, n (%)	9/120 (7.5)	2/52 (3.8)	7/68 (10.3)	0.296
Major vascular complication, n (%)	12/120 (10.0)	4/52 (7.7)	8/68 (11.8)	0.461
Major bleeding, n (%)	13/120 (10.8)	4/52 (7.7)	9/68 (13.2)	0.333
Acute renal failure, n (%)	25/120 (20.8)	12/52 (23.1)	13/68 (19.1)	0.597
Device success, n (%)	112/122 (91.8)	51/53 (96.2)	61/69 (88.4)	0.184
New pacemaker implantation, n (%)	33/120 (27.5)	8/52 (15.4)	25/68 (36.8)	0.009
Residual aortic regurgitation ≥ 2 at discharge, n (%)	7/113 (6.2)	2/49 (4.1)	5/64 (7.8)	0.697
Mean residual transaortic gradient, median (IQR), mm Hg	10 (7–14) n = 104	12 (7–15) n = 44	10 (7–13) n = 60	0.186
Length of hospital stay, median (IQR), days	14 (10–23) n = 120	14 (10–25) n = 52	13 (9–23) n = 68	0.332

Values are n (%) or median (IQR). EC-IE indicates infective endocarditis induced by Enterococci; SC-IE, infective endocarditis induced by Staphylococci; COPD, chronic obstructive lung disease; IQR, interquartile range; TAVI, transcatheter aortic valve implantation. ^a^ *p* values are results of comparing EC-IE vs. SC-IE.

**Table 2:** Main clinical characteristics, management, and outcomes of IE episode.

	All Patients (n = 122)	EC-IE (n = 53)	SC-IE (n = 69)	Unadjusted *p*-Value ^a^
Time from TAVI, median (IQR), days	110 (18–375)	142 (24–416)	85 (18–291)	0.413
Early IE (within 1 year), n (%)	91/122 (74.6)	37/53 (69.8)	54/69 (78.3)	0.288
Late IE (>1 year), n (%)	31/122 (25.4)	16/53 (30.2)	15/69 (21.7)	
Very early (within one month), n (%)	39/122 (32.0)	17/53 (32.1)	22/69 (31.9)	0.982
**Initial symptoms**				
Fever, n (%)	107/121 (88.4)	45/52 (86.5)	62/69 (89.9)	0.572
Septic shock, n (%)	29/120 (24.2)	8/52 (15.4)	21/68 (30.9)	0.049
New-onset heart failure, n (%)	69/120 (57.5)	27/52 (51.9)	42/68 (61.8)	0.280
Neurological, n (%)	27/121 (22.3)	11/52 (21.2)	16/69 (23.2)	0.790
Systemic embolism, n (%)	26/121 (21.5)	11/52 (21.2)	15/69 (21.7)	0.938
Weight loss, n (%)	8/73 (11.0)	6/31 (19.4)	2/42 (4.8)	0.065
**Echocardiographic findings**				
Vegetation, n (%)	89/116 (76.7)	40/52 (76.9)	49/64 (76.6)	0.964
Vegetation size, mm	8 (4–15) n = 46	9 (5–13) n = 22	7 (1–19) n = 24	0.895
Perivalvular extension, n (%)	19/122 (15.6)	10/53 (18.9)	9/69 (13.0)	0.379
Valve involved				
Isolated or involved THV	63/122 (51.6)	33/53 (62.3)	30/69 (52.2)	0.040
Mitral valve	19/122 (15.6)	7/53 (13.2)	12/69 (17.4)	0.528
Isolated PM	7/122 (5.7)	0/53 (0)	7/69 (10.1)	0.018
New aortic regurgitation, n (%)	7/122 (5.7)	4/53 (7.5)	3/69 (4.3)	0.466
New mitral regurgitation, n (%)	23/122 (18.9)	10/52 (18.9)	13/69 (18.8)	0.997
**Causative microorganisms**				
*Staphylococcus aureus*, n (%)			45/69 (65.2)	N/A
*Methicillin-resistant*			7/69 (10.1)	N/A
Coagulase-negative staphylococci, n (%)			17/69 (24.6)	N/A
Enterococci, n (%)		53/53 (100)		N/A
**Complications during IE hospitalization**				
Any complication, n (%)	100/122 (82.0)	37/53 (69.8)	63/69 (91.3)	0.002
Heart failure, n (%)	71/120 (59.2)	24/52 (46.2)	47/68 (69.1)	0.011
Acute renal failure, n (%)	65/118 (55.1)	21/52 (40.4)	44/66 (66.7)	0.004
Septic shock, n (%)	53/120 (44.2)	15/52 (28.8)	38/68 (55.9)	0.003
Stroke, n (%)	8/120 (6.7)	2/52 (3.8)	6/68 (8.8)	0.463
Systemic embolization, n (%)	14/120 (11.7)	7/52 (13.5)	7/68 (10.3)	0.592
Persistent bacteremia, n (%)	36/70 (51.4)	12/30 (40.0)	24/40 (60.0)	0.098
**Management and Outcomes**				
Antibiotic treatment alone, n (%)	95/122 (78.7)	42/53 (79.2)	53/69 (76.8)	0.748
Antibiotic + Surgery during IE hospitalization, n (%)	27/122 (22.1)	11/53 (20.8)	16/69 (23.2)	
Time to surgery, median (IQR), days	10 (4–35)	10 (5–48)	11 (2–23)	0.099
Open heart surgery, n (%)	22/122 (18.0)	11/53 (20.8)	11/69 (15.9)	0.120
Isolated pacemaker extraction, n (%)	5/122 (4.1)	0/53 (0)	5/69 (7.2)	
Follow-up, median (IQR), days ^b^	394 (142–980)	729 (222–1163)	292 (104–778)	0.044
In-hospital mortality, n (%)	57/120 (47.5)	19/52 (36.5)	38/68 (55.9)	0.035
1-year mortality rate, % (95% CI) ^c^	61.5 (52.2–70.1)	50.9 (36.8–64.9)	69.6 (57.3–80.0)	0.009 ^d^
Overall mortality, n (%)	87/122 (71.3)	33/53 (62.3)	54/69 (78.3)	0.053

Values are n (%) or median (IQR). EC-IE indicates infective endocarditis induced by Enterococci; SC-IE, infective endocarditis induced by Staphylococci; IQR, interquartile range; THV, transcatheter heart valve. ^a^ *p* values are results of comparing EC-IE vs. SC-IE. ^b^ Patients who survived in-hospital period. ^c^ Kaplan–Meier estimates. ^d^ Log-rank test.

**Table 3:** Factors associated with *Enterococcus* as causative microorganism in patients with IE post-TAVI.

	Univariate Analysis OR (95% CI)	Unadjusted *p* Value	Multivariate Analysis OR (95% CI)	Adjusted *p* Value
**Baseline and TAVI features**				
Age	1.05 (0.99–1.12)	0.124	1.03 (0.90–1.18)	0.688
Male gender	0.64 (0.31–1.32)	0.224	0.21 (0.04–1.00)	0.050
Body mass index > median 27.3 kg/m^2^	2.85 (1.35–5.99)	0.006	4.90 (1.19–20.16)	0.028
Atrial fibrillation	2.68 (1.28–5.61)	0.009	9.56 (2.17–42.17)	0.003
Orotracheal intubation	0.53 (0.25–1.13)	0.100		
New pacemaker implantation	0.31 (0.13–0.77)	0.011	0.09 (0.01–0.65)	0.018
**IE characteristics on admission**				
Septic shock	0.41 (0.16–1.01)	0.053	0.12 (0.02–0.71)	0.019
Weight loss	4.80 (0.90–25.67)	0.067		
**Structure involved**				
Involved THV	2.15 (1.03–4.46)	0.041	1.86 (0.49–7.06)	0.361
Cardiac device involvement *	0.54 (0.46–0.64)	0.018		

TAVI, transcatheter aortic valve implantation; THV, transcatheter heart valve. * excluded due to collinearity.

**Table 4:** Multivariate analysis of factors associated with 1-year mortality in *enterococci*-induced IE.

	Unadjusted Hazard Ratios	Unadjusted *p*-Value	Adjusted Hazard Ratios	Adjusted *p*-Value
**Baseline characteristics**				
Logistic EuroSCORE	1.03 (1.01–1.06)	0.018	0.94 (0.81–1.10)	0.449
**Initial symptoms**				
New onset heart failure ^a^	3.27 (1.40–7.62)	0.006		
Septic shock ^a^	4.96 (2.09–11.77)	<0.001		
Neurological	1.59 (0.67–3.78)	0.296		
**Echocardiography**				
THV involvement	1.06 (0.49–2.32)	0.883		
Periannular involvement	1.32 (0.76–2.31)	0.328		
**Complications during IE treatment**				
Heart failure	2.53 (1.14–5.62)	0.022	2.26 (0.37–13.96)	0.379
Acute renal failure	3.42 (1.53–7.64)	0.003	3.94 (0.70–22.26)	0.120
Septic shock	8.38 (3.67–19.15)	<0.001	6.58 (1.51–28.66)	0.012
Stroke	3.51 (0.80–15.40)	0.097		
Systemic embolization	1.15 (0.34–3.86)	0.823		
Persistent bacteremia	3.51 (1.13–10.86)	0.030	1.15 (0.26–5.05)	0.849
**Treatment**				
Cardiac Surgery	1.28 (0.51–3.17)	0.600		

THV indicates transcatheter heart valve; IE, infective endocarditis. ^a^ excluded to avoid collinearity.

**Table 5:** Multivariate analysis of factors associated with 1-year mortality in *staphylococci* induced IE.

	Unadjusted Hazard Ratios	Unadjusted *p*-Value	Adjusted Hazard Ratios	Adjusted *p*-Value
**Baseline characteristics**				
Logistic EuroSCORE	1.02 (0.99–1.04)	0.149		
**Initial symptoms**				
New onset heart failure	1.40 (0.77–2.56)	0.269		
Septic shock	1.27 (0.70–2.32)	0.429		
Neurological	1.70 (0.90–3.21)	0.105		
**Echocardiography**				
THV involvement	1.17 (0.67–2.08)	0.574		
Periannular involvement	1.08 (0.65–1.78)	0.770		
**Complications during IE hospitalization**				
Heart failure	1.40 (0.74–2.65)	0.302		
Acute renal failure	2.25 (1.14–4.47)	0.020	1.42 (0.51–3.94)	0.500
Septic shock	5.59 (2.83–11.02)	<0.001	2.27 (0.72–7.21)	0.091
Stroke	1.34 (0.53–3.40)	0.535		
Systemic embolization	1.28 (0.54–3.02)	0.574		
Persistent bacteremia	6.59 (2.22–19.53)	0.001	3.66 (1.03–13.02)	0.045
**Treatment**				
Cardiac Surgery	0.69 (0.34–1.38)	0.291		

THV indicates transcatheter heart valve; IE, infective endocarditis.

## Data Availability

The data underlying this paper will be shared upon reasonable request to the corresponding author and authors of each participating center.
